# Diversity, Mutation and Recombination Analysis of Cotton Leaf Curl Geminiviruses

**DOI:** 10.1371/journal.pone.0151161

**Published:** 2016-03-10

**Authors:** Huma Saleem, Nazia Nahid, Sara Shakir, Sehrish Ijaz, Ghulam Murtaza, Asif Ali Khan, Muhammad Mubin, Muhammad Shah Nawaz-ul-Rehman

**Affiliations:** 1 Virology Lab, Center for Agricultural Biochemistry and Biotechnology, University of Agriculture, Faisalabad, Pakistan; 2 Department of Bioinformatics and Biotechnology, GC University Faisalabad, Faisalabad, Pakistan; Oklahoma State University, UNITED STATES

## Abstract

The spread of cotton leaf curl disease in China, India and Pakistan is a recent phenomenon. Analysis of available sequence data determined that there is a substantial diversity of cotton-infecting geminiviruses in Pakistan. Phylogenetic analyses indicated that recombination between two major groups of viruses, cotton leaf curl Multan virus (CLCuMuV) and cotton leaf curl Kokhran virus (CLCuKoV), led to the emergence of several new viruses. Recombination detection programs and phylogenetic analyses showed that CLCuMuV and CLCuKoV are highly recombinant viruses. Indeed, CLCuKoV appeared to be a major donor virus for the coat protein (CP) gene, while CLCuMuV donated the Rep gene in the majority of recombination events. Using recombination free nucleotide datasets the substitution rates for CP and Rep genes were determined. We inferred similar nucleotide substitution rates for the CLCuMuV-Rep gene (4.96X10^-4^) and CLCuKoV-CP gene (2.706X10^-4^), whereas relatively higher substitution rates were observed for CLCuMuV-CP and CLCuKoV-Rep genes. The combination of sequences with equal and relatively low substitution rates, seemed to result in the emergence of viral isolates that caused epidemics in Pakistan and India. Our findings also suggest that CLCuMuV is spreading at an alarming rate, which can potentially be a threat to cotton production in the Indian subcontinent.

## Introduction

Cotton leaf curl geminiviruses (CGs) are single-stranded DNA viruses in the family *Geminiviridae* [1]. The genomes of CGs are ~2.8Kb, associated with a pathogenicity determinant molecule, namely betasatellite, and a non-essential alphasatellite [2]. Both satellites are half the size of the viral genome. Single stranded DNA viruses (family *Geminiviridae*) are rapidly evolving through mutation and recombination. The majority of the published reports of new geminiviruses were based on percentage sequence identity and phylogenetic analyses. However, detailed analyses of crop specific viruses have rarely been performed. Therefore, in this report, the evolutionary rates and recombination patterns of viruses associated with cotton leaf curl disease (CLCuD) are described.

With the establishment of new taxonomical nomenclature for geminiviruses, the begomoviruses that cause cotton leaf curl diseases in the Indian subcontinent are categorized into five different species: Cotton leaf curl Multan virus (CLCuMuV); Cotton leaf curl Bangalore virus (CLCuBaV); Cotton leaf curl Kokharan virus (CLCuKoV); Cotton leaf curl Allabad virus (CLCuAlV); and Cotton leaf curl Gezira virus (CLCuGeV) [3]. Currently, two different recurring begomoviruses (CLCuMuV and CLCuKoV) are associated with cotton leaf curl disease [4]. Six other geminiviruses, African cassava mosaic virus (ACMV), Chickpea chlorotic dwarf virus (CpCDV), Okra enation leaf curl virus (OEnLCV), Papaya leaf curl virus (PaLCV), Tomato leaf curl Bangalore virus (TLCBaV) and Tomato leaf curl New Delhi virus (TLCNDV) have also been identified in cotton [5–12]. Of 11 different viruses, Koch’s postulates have been established only for CLCuMuV, CLCuKoV and PaLCuV [2,13]. This list of viruses may increase in the future due to highly efficient cloning, sequencing and plant inoculation methods. Although these 11 species of begomoviruses were reported from India and Pakistan, the incidence of the species other than CLCuMuV and CLCuKoV on cotton is very rare [8,12–14]. This is likely due to accidental introduction on cotton through the whitefly vector [4].

Recombinants of CLCuKoV and CLCuMuV (including Burewala strain, Accession no:AM421522, Rajasthan strain, Accession no:JN678804 and Shadadpur strain Accession no:FN552001 strains) have been reported to cause epidemics in cotton in the past decade [8,15,16]. Viruses that were once limited to Pakistan are now found in neighboring countries. For example, the Burewala strain has spread from Pakistan to India during the epidemic [14]. In addition, current evidence also suggests that the Rajesthan strain evolved in India and then moved to Pakistan [4]. Among all cotton-infecting begomoviruses, CLCuMuV has the widest geographical distribution and is found in China, India and Pakistan [11,12,17].

As begomoviruses are single-stranded in nature, their proofreading activity during the DNA replication is compromised. Due to their single-stranded genome, begomoviruses offer an opportunity to understand mutation and recombination. The evolutionary rates estimated for East African cassava mosaic virus (EACMV), tomato yellow leaf curl virus (TYLCV) and maize streak virus (MSV) were 10^−4^ substitutions/nucleotide/year [18–20]. This evolutionary rate is closer to that of RNA viruses. Another important factor in begomovirus evolution is the absence of cross protection [10,21]. Despite the fact that several estimates for recombination and mutation rates were recorded for begomoviruses, evolutionary rates of CLCuD are poorly understood [18,22]

In this study, we have examined recombination and evolutionary rates of different begomoviruses infecting cotton in India and Pakistan. We have also generated information regarding the pattern of their geographical spread. As the betasatellites associated with CLCuD are monophyletic in nature and show very conserved sequences and the alphasatellites are not necessary for disease development. Therefore, we have not included these satellites in our analysis.

We found that CLCuMuV, CLCuKoV and CLCuAlV are closely related to each other and vary in their recombination patterns. The implications of recombination events in begomovirus’s speciation and emergence of cotton leaf curl disease is discussed.

## Materials and Methods

### Virus data set

Full-length sequences of Bhendi yellow vein mosaic viruses (BYVMV), CLCuMuV, CLCuKoV, CLCuAlV and ACMV were obtained from GenBank and their acronyms were selected according to [1]. A BLAST search was conducted for each virus and all related viruses (at the species level) were downloaded in fasta format. A single fasta file was generated for each species. Each genome was partitioned into Coat Protein (V1) and Rep (C1) for mutational analysis in BEAST. The phylogenetic trees of CP and Rep genes are presented in [10].

### Phylogenetic analysis

For phylogenetic analysis, sequences were first aligned in MEGA6 software using Clustal-W [23]. The phylogenetic tree was constructed, using the Maximum likelihood method with 1000 bootstrap replications. The branches of identical molecules or isolates within a species were converted into a colored triangle by using Tree Editor of MEGA6 [23]. The phylogenetic tree was constructed with 191 sequences (BYVMV = 74, CLCuALV = 8, CLCuMuV = 58, CLCuKoV = 40, PaLCuV = 10, ACMV = 1). For determining the percent nucleotide identity, viral sequences were aligned by Muscle in the sequence demarcation tool (SDT) program [3].

### Recombination analysis

The Recombination Detection Program (RDP-4) was used to detect likely parents and the extent of recombination in CG [24]. For recombination analysis, sequences of CLCuMuV, CLCuAlV, CLCuKoV and BYVMV were used (S1 File). The sequences were aligned in MEGA6 software and exported to the RDP4 program for recombination analysis. The default value of X-over, which includes automated GENECONV, BOOTSCAN, MaxChi, SiScan and 3SEQ, was used to estimate the recombination events (S1 Table). A cut off value of 0.05 was used as a *p-*value. The recombination events were also confirmed through phylogenetic analysis of individual genes and nucleotide alignment in MegAlign (Lasergene, DNA-STAR). Recombinants were confirmed by more than one method for authenticity.

### Mutation rate estimates

Because of the high recombination rate in full-length genomes, mutation rates were estimated for recombination-free data using CP gene sequences. Because of the high similarity between CP genes of CLCuAlV and BYVMV, both were considered as one set of sequences (S2 File). Similarly, CPs of CLCuKoV and Burewala strains were considered as one set of sequences (S3 File), as were Rep genes of CLCuMuV and Burewala strains (S4 File). The Rep gene of CLCuKoV and Rajasthan strains were identical (98%) to each other, therefore both were considered as one set of sequences (S5 File). The length of CLCuKoV-Rep is 1083 nt. Therefore, the last 25 nt of the Rep gene from CLCuKoV isolates were deleted. Other cotton infecting viruses such as CLCuBaV and PaLCuV were excluded from the analysis because fewer than 15 sequences were found in GenBank. The CP and Rep sequences were aligned and exported to the Beauti module of BEAST V1.7.4 [25] (http://beast.bio.ed.ac.uk/BEAST). The years of sampling were recorded from GenBank and manually added to the tips date module of Beauti. The analysis was run with general time-reversible (GTR) model of substitution rate measurements with gamma as a site-rate heterogeneity model. Genes were also partitioned into 3 codon positions (CP) for mutation analysis at each nucleotide position. Based on the previously used models for TYLCV and EACMV, relaxed and strict clock models with uncorrelated lognormal distribution were used [18,19]. For Rep genes, only the relaxed clock model was found to be appropriate. A uniform prior distribution was used (0–100) for all the datasets. Individual BEAST runs were performed at least 2 times with variable Markov chain lengths to ensure a higher value of the effective sample size (ESS, >100). Rep genes of CLCuKoV, CLCuMuV and CP of CLCuAlV or BYVMV were run for 10 X 10^7^ steps with the initial 10% discarded as burnin values for the relaxed clock model. CLCuKoV-CP was run for 3 X 10^8^ steps with 10% burn-in value for the relaxed clock model. Each BEAST analysis was viewed in Tracer V1.6 (http://beast.bio.ed.ac.uk/Tracer).

## Results

### Two centers of diversity for cotton infecting Geminiviruses

**Fig 1:**
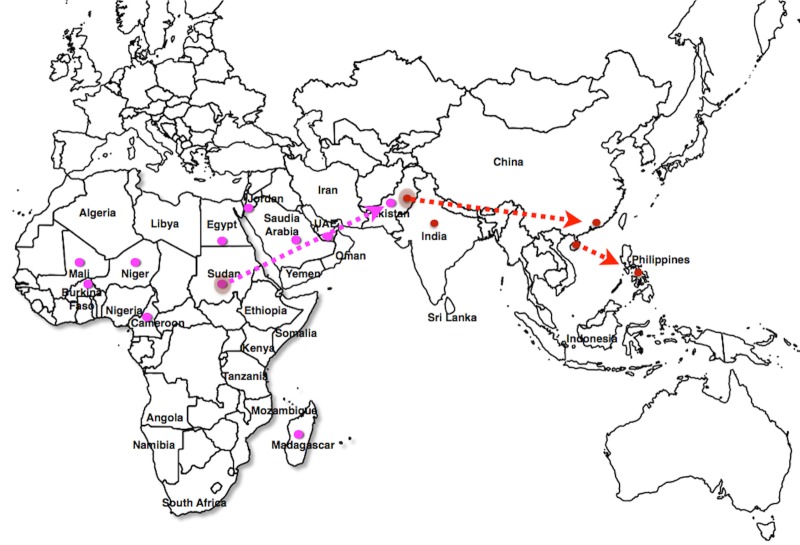
Pakistan and India are centers for diversity for cotton leaf curl disease in Asia. The disease was first reported in Pakistan and later appeared in India and China. Introduction of Cotton leaf curl Multan virus to the Philippines is a recent occurence and marks the capability of the disease to spread in very far off places from the center of diversity. The major areas in which CLCuMuV was found are shown with red circles. CLCuGeV is spreading worldwide through infection of cotton and okra plants. Currently, it is found in 11 countries (marked with pink colored circles) and is spreading from North Africa to rest of the world.

**Fig 2:**
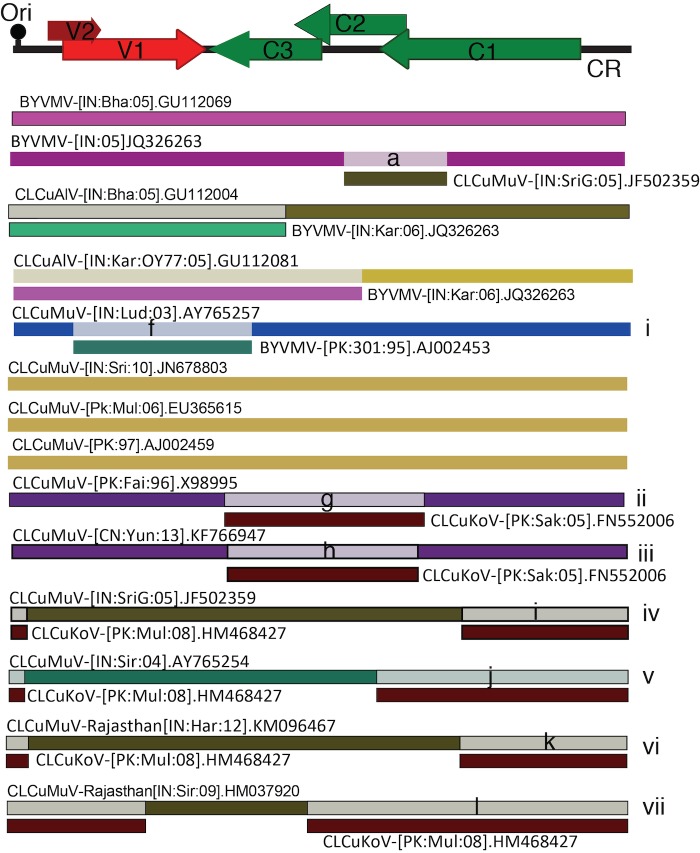
Recombination analyses of cotton leaf curl geminiviruses with the recombination detection program (RDP-4). The diversification of viruses infecting cotton occurred from recombination of Cotton leaf curl Multan virus (CLCuMuV) and Cotton leaf curl Kokharan virus (CLCuKoV). CLCuMuV-AY765254, Shadadpur strain, Burewala strain, and Rajasthan strains are mainly recombinants for CLCuKoV and CLCuMuV. However, cotton leaf curl Alabad virus (CLCuAlV) and CLCuMuV-AY765257 are recombinants between Bhendi yellow vein mosaic virus (BYVMV) and an unknown parent.

Eleven different viruses were associated with cotton plants (n = 11) in China, India, Pakistan and Africa (Fig 1). In Pakistan and India, almost equal numbers of viruses were found on cotton plants (Table 1). CLCuGeV and ACMV, which are viruses of African origin [26,27], were only found in Pakistan [10]. Similarly, Chickpea chlorotic dwarf virus (CpCDV), which belongs to genus *Mastrevirus*, was also found on cotton in Pakistan [28]. Although, CpCDV has been reported in India, until now it was not reported to infect cotton [29]. ACMV and CpCDV were found in mixed infection with other begomoviruses in Pakistan. Therefore, it cannot be concluded that they alone have a potential to cause the leaf curl disease in cotton. According to the information available in GenBank, CLCuMuV has been found in okra, cotton and hibiscus plants in southeastern China (Fig 1), while in the Philippines, CLCuMuV has been found only on hibiscus plants. The Chinese isolates show relatively higher sequence identity %age (91–96%) with the Multan isolates from Pakistan as compared to those found in India (88.2–95%) (Table 2). The deeper genome analysis through recombination (Fig 2) showed that the Faisalabad strain (Accession no, X98995) is indeed present in China, India, Pakistan and the Philippines. This supports the possible introduction of CLCuMuV from Pakistan to China and Philippines (Fig 1). Similarly, isolates identified from the Philippines show high percentage identity with Chinese isolates (98.9–99.3%), clearly indicating that CLCuMuV in the Philippines was introduced through infected hibiscus plants in recent years from Southeastern China or Pakistan. Because of the greater diversity and higher number of geminiviruses found in cotton in India and Pakistan, we concluded that this region is the center for diversity of CGs in Asia.

**Table 1:** Presence of different viruses related to cotton leaf curl disease in various geographical locations.

Ser#	Virus Name	Pakistan	India	Philippines*	China	References
1	CLCuMuV	Yes	Yes	Yes	Yes	[4,11,13,14,16]
2	CLCuKoV	Yes	Yes	No	No	[7,13,15]
3	CLCuGeV	Yes	No	No	No	[30]
4	CLCuAlV	Yes	Yes	No	No	[10,12]
5	ACMV	Yes	No	No	No	[10]
6	OLCEnV	Yes	No	No	No	[6]
7	ToLCNDV	No	Yes	No	No	Accession No. EF063145
8	ToLCBaV	No	Yes	No	No	[7]
9	CLCuBaV	No	Yes	No	No	[5]
10	CpCDV	Yes	No	No	No	[9]
11	PaLCV	Yes	Yes	No	No	[12,37]

* The viruses found in the Philippines were reported from hibiscus instead of cotton.

**Table 2:** Nucleotide identity percentages for Multan isolates from Asia.

	CLCuMuV-China	CLCuMuV-Philippines	CLCuMuV-Pakistan	CLCuMuV-India
**CLCuMuV-China**	99.2–99.8	98.9–99.3	91–96	88.2–95
**CLCuMuV-Philippines**		99	91.7–95.8	91–94.6
**CLCuMuV-Pakistan**			91–99.9	89.4–99.7
**CLCuMuV-India**				87–98

The spread of CLCuGeV to different geographical areas is alarming. With the current available data, CLCuGeV is present in at least 11 different countries (Fig 1). Apparently, the spread occurred from North Africa to West Africa, and from the Middle East to South Asia [30]. Unlike CLCuMuV, which possibly spread through hibiscus plants, CLCuGeV seems to require its whitefly vector. Recently, CLCuGeV was identified in Okra plants in Saudi Arabia and United Arab Emirates (UAE). Therefore, it is possible that CLCuGeV has spread from North Africa to Pakistan through Saudi Arabia and UAE.

### Phylogeny of CG displays a complex inter- and intra-species recombination

As recombination is a major factor in geminivirus evolution, we compared 191 full-length genomes of CGs (including BYVMV). Interestingly, recombination was observed mainly between CLCuMuV and CLCuKoV with the exception of CLCuAlV (Fig 2). Newly identified strains, Rajasthan, Burewala and Shadadpur were recombinants of these two viruses [7,10,15,16]. However, they varied in the number of nucleotides spanned by both the parents. Another important recombination event in CGs was between BYVMV and CLCuMuV (Fig 2). For CLCuAlV, the viral genes in virion strands were derived from BYVMV, while the Rep associated region was derived from CLCuMuV.

**Fig 3:**
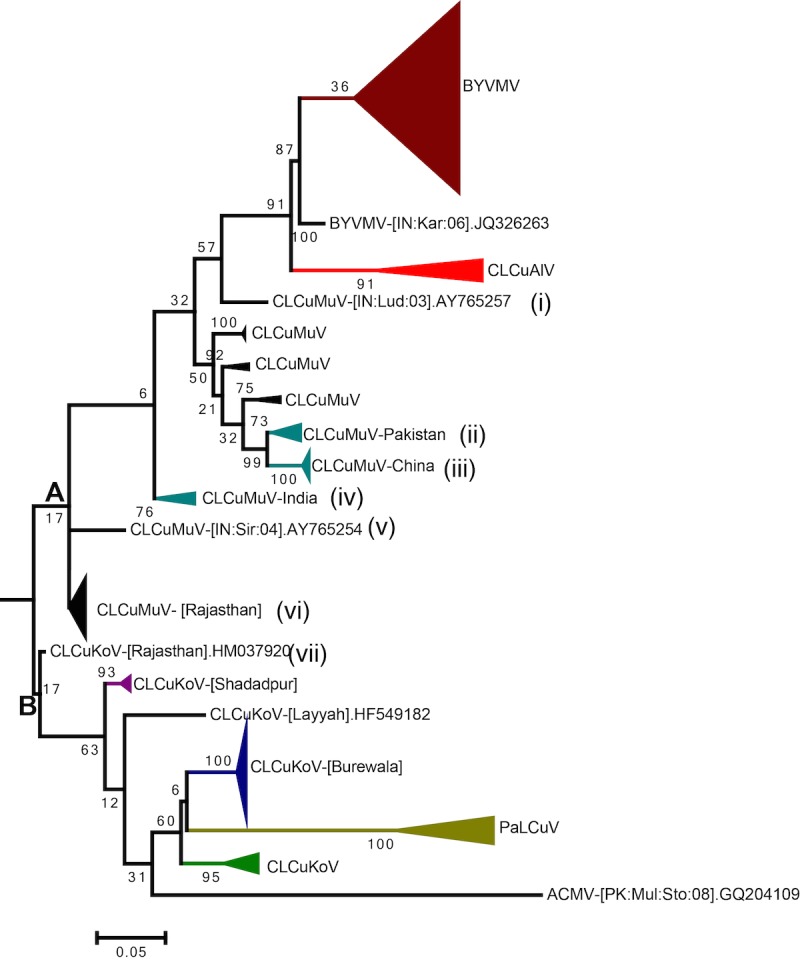
Maximum likelihood phylogenetic tree of full-length cotton leaf curl geminiviruses: The phylogenetic tree was generated for cotton-infecting begomoviruses. Group-A represents the major cotton-infecting viruses whose sequences are mainly derived from CLCuMuV. Group-B represents viruses whose sequences are mainly derived from CLCuKoV. Seven different clades are found because of recombination between CLCuMuV and CLCuKoV, or BYVMV. Each clade shows a specific recombination pattern (see Fig 2). African cassava mosaic virus was chosen as an out-group in the phylogenetic analysis. The numbers at the nodes represent bootstrap values.

Although a list of 11 different begomoviruses is presented in Table 1, only Burewala, Rajasthan and Shadadpur strains are found in routine surveys. The extent of recombination represents different clades in the phylogenetic tree (Fig 3). The phylogenetic tree depicts CGs falling into two main groups: group-A represents the recombinant viruses mainly derived from CLCuMuV and group-B represents the viruses derived by recombination of CLCuKoV. Group-A also included viruses derived from recombination between CLCuKoV and CLCuMuV (Rajasthan strains and the isolate AY765254). However, since the region spanned by CLCuMuV sequences is greater than CLCuKoV, they are presented in group-A. CLCuMuV-AY765257 (clade-i) makes a basal branch in BYVMV and CLCuAlV clades, with recombination analysis revealing the presence of BYVMV sequences in the CP region of this isolate (Fig 2).

A unique phylogenetic pattern is observed for CG in the phylogenetic tree. The clade-ii isolates represent the Multan isolates, which have short stretches of C2/C3 and C1 genes from CLCuKoV (Fig 3). A similar recombination pattern is observed in the Chinese and Filipino isolates (Fig 3, clade-iii).

The Indian isolates of CLCuMuV also show a unique recombination pattern as compared with the Chinese isolates. In Indian isolates, CLCuKoV donated the Rep gene, common region, and origin of replication to the Multan isolates. All isolates of the Rajasthan strain are recombinants between CLCuMuV and CLCuKoV, with varying lengths of CLCuKoV sequences (Clades-v and vi in Figs 2 and 3). Clade-vii is marked by a single isolate (HM037920), which spans virion and complementary strands nucleotides from CLCuKoV, while CLCuMuV shares a short stretch of the V1 region. Due to more nucleotides shared by CLCuKoV, this isolate is closer to group-B.

### CGs have variable mutation rates

To estimate the mutation rate, we used the recombination free data set for the CP and Rep genes. Mutation analysis for CLCuMuV was published previously [10]. The mean substitution rate calculated for CLCuAlV/BYVMV was 4.761 X 10^−4^ and 7.265 X 10^−4^ substitution/nt/year using both strict and relaxed clock models, respectively. Burewala, Shadadpur strains and CLCuKoV share CP [15,16], thus they were included in our mutation rate estimate and were mainly presented as CLCuKoV (Table 3). With the strict clock model, the mean evolutionary rate for the CLCuKoV-CP gene was estimated as 9.70 X 10^−5^ substitutions/nt/year, while with the relaxed clock model, the mean rate was 2.706 X 10^−4^ substitutions/nt/year. This showed that the mean substitution rate for the CP gene of CLCuKoV is relatively low compared to CLCuMuV (4.24 X 10^−4^ substitutions/nt/year). However, the substitution rates for the CP gene of CLCuKoV were within the range of previously known substitution rates for EACMV and TYLCV (Table 3). The mean substitution rate for Rep genes of CLCuKoV was relatively higher (9.05 X 10^−4^, with 95% HPD of 3.96 X 10^−4^ to 1.45 X 10^−3^) compared with CLCuMuV (4.96 X 10^−4^, with 95% HPD 7.64 X 10^−4^–2.45 X 10^−4^).

**Table 3:** Evolutionary rate analysis of cotton leaf curl geminiviruses.

Virus name	Demographic/clock model	Mean Rate	95% High posterior density (HPD)	CP1^!^ Mutation rate	CP2 Mutation rate	CP3 Mutation rate	No of Taxa
CLCuAlV/BYVMV-CP	GTR+Gamma/Strict	4.761X10^-4^	1.65X10^-4^–7.85X10^-4^	0.306	0.289	2.405	77
CLCuAlV/BYVMV-CP	GTR+Gamma/Relaxed	7.265X10^-4^	1.40X10^-4^–1.35X10^-3^	0.289	0.272	2.439	77
CLCuKoV-CP	GTR+Gamma/Strict	9.70X10^-5^	6.62X10^-6^–1.35X10^-4^	0.54	0.85	1.61	44
CLCuKoV-CP	GTR+Gamma/Relaxed	2.706X10^-4^	2.801X10^-6^–1.207X10^-3^	0.54	0.85	1.61	44
CLCuKoV-Rep	GTR+Gamma/Relaxed	9.05X10^-4^	3.96 X 10^−4^–1.45X10^-3^	0.924	0.724	1.35	30
CLCuMuV-Rep	GTR+Gamma/Relaxed	4.96X10^-4^	7.64X10^-4^–2.45X10^-4^	0.821	0.572	1.607	53
CLCuMuV-CP*	BSP/Relaxed	4.24X10^-4^	-	1.64	0.449	0.909	19
GDarSLA-Rep*	BSP/Relaxed	2.13X10^-3^	-	1.4	0.765	0.831	63
CLCuMuB- βC1*	BSP/Relaxed	3.51X10^-3^	-	0.85	0.73	1.43	39
EACMV-CP**	BSP/Relaxed	1.37X10^-3^	-	-	-	-	71
TYLCV-CP***	Relaxed+Exponential	4.63X10^-4^	-	-	-	-	54

! codon position (CP)

* Nawaz-ul-Rehman et al., 2012.

** Duffy and Holmes, 2009.

*** Lefeuvre et.al 2010.

### Substitution bias in codon positions of CG genes

The mutation estimates at three different codon positions of CLCuKoV and CLCuAlV-CP showed similar substitution patterns. Maximum substitution rate was observed for the third or wobble codon position with a mean substitution rate of 2.4 and 1.6 for CLCuAlV and CLCuKoV, respectively while codon positions 1 and 2 have considerably lower mean substitution rates (Table 2). The Rep gene of CLCuKoV showed a relatively high mean substitution rate at the first codon position (0.924). The value of the first codon position was closer to the third codon position (1.35). Similar results were observed for the CP gene of CLCuMuV (1.6 at codon position 1). For CLCuMuV, the mean substitution rate at the first codon position in the Rep gene was considerably lower (0.821) compared to the first codon position of the CP gene.

## Discussion

CLCuD has been prevalent in Pakistan for almost half a century [4,31], while in Africa it has been known to exist for hundreds of years [32]. Although there has been only a single species known in Africa, more than a dozen strains are known to be present in cotton in Asia. Among these viruses, the Burewala strain is the most dominant in India and Pakistan. Most surprisingly, CLCuMuV is now found in Southeastern China and the Philippines, possibly because Southeastern China is affiliated with more migration and trade compared to the rest of the country. The role of the whitefly in local transmission of viruses cannot be ignored. Based on the current data, it is clear that whitefly has played a significant role in CLCuD epidemics in India and Pakistan [33,34]. However, there are no data available, suggesting the movement of whitefly from Pakistan to China or the Philippines. Since, hibiscus is propagated through stem cuttings, it is possible that CLCuMuV was introduced into Southeastern China through trade of hibiscus plants. The Chinese and Philippine isolates of CLCuMuV form a monophyletic group in the phylogenetic tree, but cluster with Pakistani isolates. The Philippines is geographically isolated from the rest of Asia with no land connections and does not share a border with countries where CLCuMuV is present. CLCuMuV has only been isolated from hibiscus in the Philippines. Thus it can be hypothesized that it is a recent introduction from Southeastern China.

CLCuMuV, CLCuKoV, Burewala strain, Rajesthan strain and Shadadpur strain are frequently found on cotton plants. Our analysis indicates that diversification of cotton viruses occurred because of recombination between CLCuMuV and CLCuKoV. However, there are few strains that are recombinants between CGs and BYVMV. Nonetheless, a consensus exists that several types of recombinants are found between CLCuMuV and CLCuKoV. These recombinants either exchanged complementary sense genes or virion strand genes and vice versa. For the Burewala strain, the most successful event was the exchange of virion strand genes between CLCuMuV and CLCuKoV. In other words, there has been a positive selection for CLCuKoV-CP and the Rep gene of CLCuMuV.

A substitution bias clearly exists in mutation of different codon positions for the CP and Rep genes of both the viruses. There is a high mutation frequency at the first codon position in the CP gene of CLCuMuV and the Rep gene of CLCuKoV. The high mutation load may represent negative selection pressure for the parental strains. Despite extensive surveys for CLCuD, both parental strains are rarely found in Pakistan [16]. In GenBank, there are only 6 accessions for CLCuKoV, while 50 accessions of CLCuMuV are available. Interestingly, CLCuMuV is nowadays found only in territories where recombinants have not been reported so far. For example, CLCuMuV was recently found in China [17] and the Philippines (Accessions, KF413618 and KF413616 in GenBank). It is unclear why only CLCuMuV has been found on hibiscus in different countries, while the highly epidemic Burewala strain is only present in Pakistan and India. Similarly, BYVMV has also been found in many countries, including Pakistan, India, Bangladesh and Thailand [12,35,36].

Our analysis revealed that CGs have high substitution rates, just like other known geminiviruses infecting tomatoes and cassava [18,19]. Since fewer sequences were available for CLCuKoV, we chose the CP gene of Burewala as the dataset for CLCuKoV. A similar approach was used for the CP dataset for CLCuAlV and BYVMV. In the same way, the Rep genes of CLCuBuV and CLCuMuV were collectively used for estimating their substitution rate. The analyzed data was sampled over a 17-year period (1997–2013), which provided sufficient dated sequences to infer substitution rates. The CP genes of CLCuAlV and BYVMV showed mean substitution rates of 4.761 X 10^−4^ and 7.265 X 10^−4^ with a narrow 95% HPD interval (Table 3). In comparison with BYVMV-CP, CLCuMuV-CP and EACMV-CP, the mean substitution rate of CLCuKoV-CP was relatively low using the relaxed clock model (9.70 X 10^−5^). CLCuMuV-Rep gene analysis revealed a similar substitution rate (4.96 X 10^−4^) as reported for TYLCV (4.63 X 10^−4^), using a relatively narrow 95% HPD interval (Table 3). We concluded that the CP gene of CLCuKoV and the Rep protein gene of CLCuMuV have relatively low substitution rates, which retain less mutation load. Since geminviruses exhibit no cross protection like RNA viruses, recombination occurs during mixed infections. We hypothesize that, due to natural selection, only recombinants between CLCuKoV and CLCuMuV survive. These recombinants may have strong replication ability with less mutation load. The evolution of viral genomes is believed to be the result of natural selection, virus-vector interaction, virus host interaction, and factors affecting recombination and nucleotide substitution. Our analysis of different cotton leaf curl geminiviruses reveals that recombination is an important process, since it involves the combination of divergent genetic backgrounds conferring resistance breakdown and adaptation to vector. It can be hypothesized that success of recombinants is dependent upon a balanced nucleotide substitution rate.

Currently, the Rajesthan strain is the only strain where CLCuKoV contributes the Rep gene. It is proposed that CLCuRaV originated in India and was later introduced in Pakistan [4]. Since CLCuBuV is a dominant virus in both Pakistan and India, we suggest that the most infectious combination is the presence of complementary strand genes from CLCuMuV and virion strand gene from CLCuKoV.

**Fig 4:**
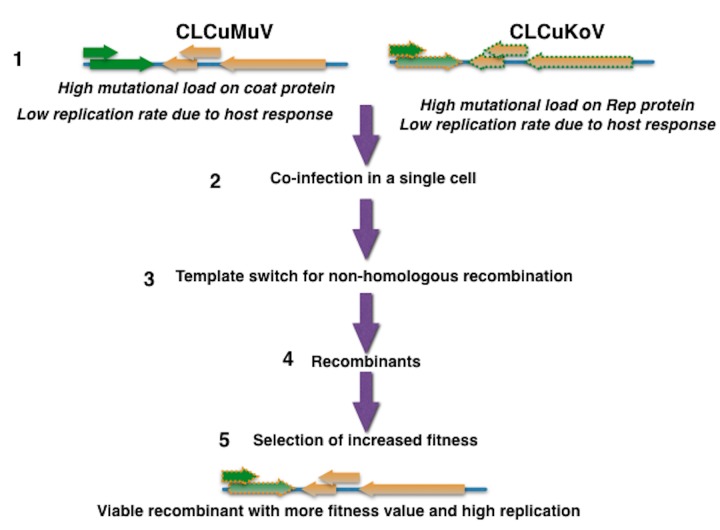
Predicted model for cotton leaf curl geminivirus evolution. The spread of CLCuDV occured due to recombination. Recombinant viruses have the advantage of low mutational load (see text) compared to parental viruses. The presence of recombinants indicates mixed infections and no cross protection among geminiviruses. The pattern of recombination suggests that several combinations are possible. However, not all recombinants have the potential to cause epidemics. The epidemic of CLCuD occurred due to a perfect combination and positive selection of recombinants between CLCuMuV and CLCuKoV.

The current scenario of cotton leaf curl disease epidemic suggests that there is a need for strict quarantine measures for movement of ornamental or other susceptible plants, which can potentially harbor begomoviruses. However, the role of the whitefly also cannot be ignored. There is a need to ensure the timely diagnosis and implementation of control measures before the disease spreads on a large scale. Particularly, all efforts should be focused on controlling CLCuMuV spread in China and the Philippines. Our analysis indicates that success of recombinant viruses is not just random. Rather, it is a combination of favorable sequences, which are more stable and can cause epidemics. On the basis of these observations, we present a model that explains the reason for the spread of recombinants in cotton (Fig 4). In conclusion, the large-scale spread of CLCuD occurred due to combinations of those genes that have low mutation rates.

## Supporting Information

S1 File Fasta file of all the sequences used for recombination analysis in recombination detection program (RDP). (FAS)

S2 File The CP gene of CLCuAlV and BYVMV used in BEAST analysis. (XML)

S3 File The CP gene of CLCuKoV used in BEAST analysis. (XML)

S4 File The Rep gene of CLCuMuV used in BEAST analysis. (XML)

S5 File The Rep gene of CLCuKoV used in BEAST analysis. (XML)

S1 Table Average, p, values of recombination events in Fig 2. (DOCX)
