# Supplementary material for: Diversity, Mutation and Recombination Analysis of Cotton Leaf Curl Geminiviruses
Source: PLoS One. 2016 Mar 10;11(3):e0151161. doi: 10.1371/journal.pone.0151161 (PMC4872795; doi:10.1371/journal.pone.0151161)
Supplement: S1 Table — (DOCX) [file pone.0151161.s006.docx]

| **Recombination event** | **Method** | **Average p values** |
| --- | --- | --- |
| a | RDP | 1.83 X 10^-14^ |
|  | GENECONV | 5.356 X 10^-17^ |
|  | Max Chi | 7.056 X 10^-12^ |
|  | Chimaera | 3.745 X 10^-11^ |
|  | SiScan | 6.767 X 10^-14^ |
| b | RDP | 1.005 X 10^-11^ |
|  | GENECONV | 2.303 X 10^-09^ |
|  | Chimaera | 9.789 X 10^-03^ |
|  | SiScan | 1.031 X 10^-05^ |
| c | RDP | 1.488 X 10^-01^ |
|  | GENECONV | 1.117 X 10^-06^ |
|  | Chimaera | 4.245 X 10^-04^ |
|  | SiScan | 5.574 X 10^-12^ |
| d | RDP | 9.994 X 10^-01^ |
|  | Max Chi | 7.433 X 10^-04^ |
| e | RDP | 1.831 X 10^-14^ |
|  | GENECONV | 5.356 X 10^-17^ |
|  | Max Chi | 7.056 X 10^-12^ |
|  | Chimaera | 3.745 X 10^-11^ |
|  | SiScan | 6.767 X 10^-14^ |
| f | RDP | 6.251 X 10^-30^ |
|  | GENECONV | 1.563 X 10^-25^ |
|  | Max Chi | 2.205 X 10^-12^ |
|  | Chimaera | 2.718 X 10^-13^ |
|  | SiScan | 1.090 X 10^-15^ |
| g | RDP | 6.380 X 10^-18^ |
|  | GENECONV | 1.401 X 10^-25^ |
|  | Max Chi | 1.094 X 10^-08^ |
|  | SiScan | 2.554 X 10^-17^ |
| h | RDP | 1.993 X 10^-37^ |
|  | GENECONV | 5.356 X 10^-30^ |
|  | Max Chi | 3.706 X 10^-20^ |
|  | Chimaera | 1.609 X 10^-20^ |
|  | SiScan | 6.231 X 10^-33^ |
| i | RDP | 1.993 X 10^-37^ |
|  | GENECONV | 5.356 X 10^-30^ |
|  | Max Chi | 3.706 X 10^-20^ |
|  | Chimaera | 1.609 X 10^-20^ |
|  | SiScan | 6.231 X 10^-33^ |
| j | RDP | 1.993 X 10^-37^ |
|  | GENECONV | 5.356 X 10^-30^ |
|  | Max Chi | 3.706 X 10^-20^ |
|  | Chimaera | 1.609 X 10^-20^ |
|  | SiScan | 6.231 X 10^-33^ |
| k | RDP | 3.159 X 10^-08^ |
|  | GENECONV | 2.027 X 10^-09^ |
|  | Max Chi | 2.162 X 10^-06^ |
|  | Chimaera | 1.166 X 10^-09^ |
|  | SiScan | 4.727 X 10^-11^ |
| l | RDP | 3.159 X 10^-08^ |
|  | GENECONV | 2.027 X 10^-09^ |
|  | Max Chi | 2.162 X 10^-06^ |
|  | Chimaera | 1.166 X 10^-09^ |
|  | SiScan | 4.727 X 10^-11^ |

Supplementary table-1. Average p values of recombination events in figure-2.
